# Myelination in the absence of UDP-galactose:ceramide galactosyl-transferase and fatty acid 2 -hydroxylase

**DOI:** 10.1186/1471-2202-12-22

**Published:** 2011-03-02

**Authors:** Marion Meixner, Julia Jungnickel, Claudia Grothe, Volkmar Gieselmann, Matthias Eckhardt

**Affiliations:** 1 Institute of Biochemistry and Molecular Biology, University of Bonn, Germany; 2 Institute of Neuroanatomy, Medical School Hannover, Germany

## Abstract

**Background:**

The sphingolipids galactosylceramide (GalCer) and sulfatide are major myelin components and are thought to play important roles in myelin function. The importance of GalCer and sulfatide has been validated using UDP-galactose:ceramide galactosyltransferase-deficient (*Cgt*^-/-^) mice, which are impaired in myelin maintenance. These mice, however, are still able to form compact myelin. Loss of GalCer and sulfatide in these mice is accompanied by up-regulation of 2-hydroxylated fatty acid containing (HFA)-glucosylceramide in myelin. This was interpreted as a partial compensation of the loss of HFA-GalCer, which may prevent a more severe myelin phenotype. In order to test this hypothesis, we have generated *Cgt*^-/- ^mice with an additional deletion of the fatty acid 2-hydroxylase (*Fa2h*) gene.

**Results:**

*Fa2h*^-/-^/Cgt^-/- ^double-deficient mice lack sulfatide, GalCer, and in addition HFA-GlcCer and sphingomyelin. Interestingly, compared to *Cgt*^-/- ^mice the amount of GlcCer in CNS myelin was strongly reduced in *Fa2h*^-/-^/*Cgt*^-/- ^mice by more than 80%. This was accompanied by a significant increase in sphingomyelin, which was the predominant sphingolipid in *Fa2h*^-/-^/*Cgt*^-/- ^mice. Despite these significant changes in myelin sphingolipids, compact myelin was formed in *Fa2h*^-/-^/*Cgt*^-/- ^mice, and g-ratios of myelinated axons in the spinal cord of 4-week-old *Fa2h*^-/-^/*Cgt*^-/- ^mice did not differ significantly from that of *Cgt*^-/- ^mice, and there was no obvious phenotypic difference between *Fa2h*^-/-^/*Cgt*^-/- ^and *Cgt*^-/- ^mice

**Conclusions:**

These data show that compact myelin can be formed with non-hydroxylated sphingomyelin as the predominant sphingolipid and suggest that the presence of HFA-GlcCer and HFA-sphingomyelin in *Cgt*^-/- ^mice does not functionally compensate the loss of HFA-GalCer.

## Background

**Figure 1:**
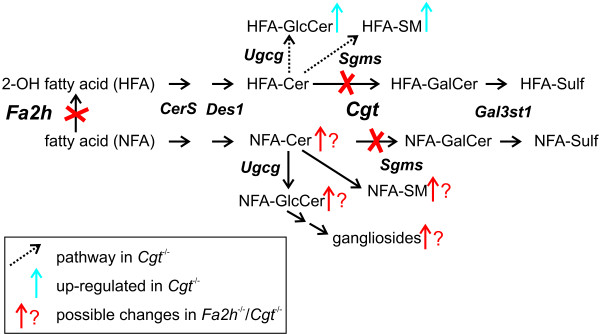
**Pathway of sphingolipid biosynthesis in myelinating glia cells**. Changes in sphingolipids observed in *Cgt*^-/- ^mice (blue arrows) and expected possible changes in *Fa2h*^-/-^/*Cgt*^-/- ^mice (red arrows). Shown are the genetic symbols of genes encoding the respective enzymes: *Cgt*, UDP-galactose:ceramide galactosyltransferase; *CerS*, ceramide synthase; *Des1*, dihydroceramide desaturase; *Fa2h*, fatty acid 2-hydroxylase; *Gal3st1*, galactose-3-O-sulfotransferase 1; *Sgms*, sphingomyelin synthase; *Ugcg*, UDP-glucose ceramide glucosyltransferase.

Galactosylceramide (GalCer) is the most abundant sphingolipid of mammalian myelin [1]. It is synthesized in the endoplasmic reticulum by UDP-galactose:ceramide galactosyltransferase (encoded by the *Cgt *gene). In the Golgi apparatus, part of the GalCer is sulfated by cerebroside sulfotransferase (encoded by the *Gal3st1 *gene), forming sulfatide [2] (see Figure 1). In the absence of a functional *Cgt *gene, compact myelin can be formed, which is, however, unstable and *Cgt*^-/- ^mice develop tremors and ataxia at 3 to 4 weeks of age [3,4]. This phenotype could be explained by disturbed axon-glial contacts at the paranodes in the CNS caused by mistargeting of essential adhesion molecules, NF-155 and Caspr [5,6]. At least in part, these structural alterations are caused by the loss of sulfatide rather than GalCer, as demonstrated by a similar alteration of the paranodal region in *Gal3st1*-deficient mice, which lack sulfatide but have normal GalCer levels [7]. However, myelin appear to be more stable in *Gal3st1*-deficient mice, suggesting additional, yet less defined roles of GalCer in myelin [8].

In *Cgt*^-/- ^mice, the loss of GalCer and sulfatide is accompanied by a significant upregulation of 2-hydroxylated fatty acid-containing (HFA) glucosylceramide (HFA-GlcCer) and HFA-sphingomyelin [3,4]. Interestingly, there are no indications for presence of HFA-gangliosides or other higher glycosylated HFA-sphingolipids in the brain of *Cgt*^-/- ^mice [9]. The presence of HFA-GlcCer and HFA-sphingomyelin was interpreted as a compensatory upregulation, which may also (in part) functionally replace HFA-GalCer, enabling *Cgt*^-/- ^mice to form compact myelin [3,10].

HFA-sphingolipids in CNS and PNS myelin are synthesized from 2-hydroxylated fatty acids, formed by the fatty acid 2-hydroxylase (encoded by the *Fa2h *gene) [11-14]. Although loss of 2-hydroxylated sphingolipids in myelin does not affect initial myelin formation, it causes late onset (in mice older than 6 months) axon and myelin sheath degeneration [15]. *Fa2h*-deficiency was identified as the cause of a new leukodystrophy with spastic paraparesis [16], and hereditary spastic paraplegia SPG35 [17], respectively.

In order to test the hypothesis that the presence of HFA-GlcCer and HFA-sphingomyelin in *Cgt*^-/- ^mice is a functional important compensatory upregulation, preventing a more severe phenotype, we generated *Cgt*^-/- ^mice with an additional deficiency in the *Fa2h *gene. Our analysis shows that the additional deletion of *Fa2h *does not obviously affect the phenotype of *Cgt^-/- ^*mice. This suggests that HFA-GlcCer and HFA-sphingomyelin do not functionally compensate the loss of HFA-GalCer in *Cgt^-/- ^*mice.

## Results

### Generation of *Fa2h*^-/-^*/Cgt*^-/- ^double deficient mice

In order to test the hypothesis that HFA-GlcCer partially compensates the loss of HFA-GalCer in *Cgt*^-/- ^mice and thereby prevents a more severe phenotype, we generated *Cgt*^-/- ^mice with an additional deficiency in *Fa2h*. As shown previously, young *Fa2h*^-/- ^mice form structural and functional normal myelin [15] and did not show behavioral abnormalities that would indicate myelin deficiency. Older *Fa2h*^-/-^, however, developed a progressive axonal degeneration in peripheral nerves and brainstem, accompanied by myelin sheath degeneration [15]. As shown before [3,4], *Cgt*^-/- ^mice had a strongly reduced life span, whereas *Fa2h*^-/- ^mice did not show increased mortality (data not shown). Survival of *Fa2h*^-/-^/*Cgt*^-/- ^double deficient mice was not significant different from *Cgt*^-/- ^mice (around 50% survival at four weeks of age). There were no obvious behavioral differences between the two genotypes. However, because of the low amount of age-/weight- and gender-matched 4-week-old *Cgt*^-/- ^and *Fa2h*^-/-^/*Cgt*^-/- ^mice available, extensive behavioral testing could not be performed, and thus minor behavioral differences between *Cgt*^-/- ^and *Fa2h*^-/-^/*Cgt*^-/- ^mice cannot be ruled out. The following biochemical and morphological analyses were done with mice at 4 weeks of age.

### GlcCer levels are reduced in CNS and PNS of *Fa2h*^-/-^/*Cgt*^-/-^mice when compared to *Cgt*^-/- ^mice

**Figure 2:**
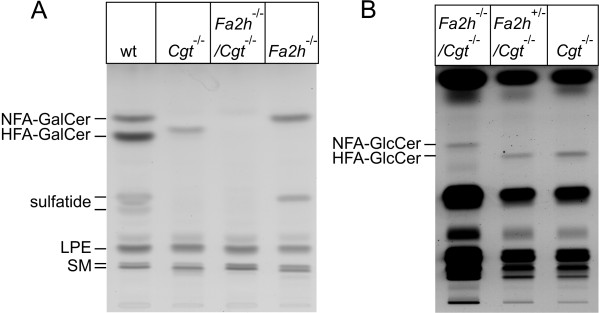
**Brain lipid analysis of *Fa2h*^-/-^/*Cgt*^-/- ^mice**. (A) TLC analysis of sphingolipids (after mild alkaline hydrolysis) from total brain of 4-week-old animals of the indicated genotypes showed a strong reduction of GlcCer in *Fa2h*^-/-^/*Cgt*^-/- ^(lane 3) compared to *Cgt*^-/- ^mice (lane 2). The amount of lipids isolated from 0.05 mg of tissue (wet weight) was loaded per lane. LPE, lyso-phosphatidylethanolamine; SM, sphingomyelin. (B) TLC analysis of total brain lipids (not subjected to alkaline hydrolysis). In order to demonstrate the presence of NFA-GlcCer in *Fa2h*^-/-^/*Cgt*^-/- ^mice, the amounts of lipids was increased 4-fold in case of *Fa2h*^-/-^/*Cgt*^-/- ^mice.

**Figure 3:**
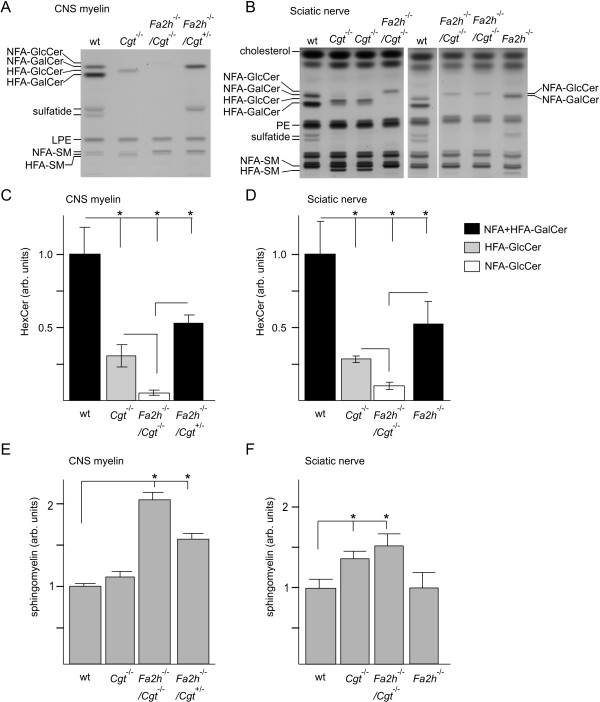
**Myelin lipid analysis of *Fa2h*^-/-^/*Cgt*^-/- ^mice**. (A) TLC analysis of lipids extracted from purified myelin confirmed the low concentration of NFA-GlcCer in *Fa2h*^-/-^/*Cgt*^-/- ^mice and showed that myelin from *Fa2h*^-/-^/*Cgt*^-/- ^mice contained larger amounts of NFA-sphingomyelin (lane 3) compared to wild-type (lane 1) or *Cgt*^-/- ^mice (lane 2). Lipid samples were subjected to alkaline hydrolysis before TLC. LPE, lyso-phosphatidylethanolamine; SM, sphingomyelin. (B) Total lipids were isolated from sciatic nerves of 4-week-old mice of the indicated genotypes and separated by HPTLC. Shown are 2 representative chromatograms (Note that lanes 5 to 8 are from the same TLC plate, however, 2 lanes containing lipids from mice with other genotypes have been removed from the Figure). PE, phosphatidylethanolamine. Hexosylceramide (HexCer) (C, D) and sphingomyelin levels (E, F) in CNS myelin (C, E) and sciatic nerves (D, F) were determined by densitometric scanning of HPTLC plates. HexCer levels were normalized to cholesterol. Data were combined from three independent experiments, and lipids from 3-6 animals per genotype were analyzed. Shown are the mean ± SD (CNS myelin: n = 3-5; sciatic nerves: n = 4-6); asterisks indicate statistically significant differences (ANOVA with post hoc Fisher's LSD test, *p < 0.05).

TLC analysis of total brain lipids from wild-type, *Fa2h*^-/-^, *Cgt*^-/- ^and *Fa2h*^-/-^/*Cgt*^-/-^mice showed significant levels of HFA-GlcCer in *Cgt*^-/-^mice, in line with earlier reports [3,4]. Unexpectedly, NFA-GlcCer levels in total brain of *Fa2h*^-/-^/*Cgt*^-/-^mice were strongly reduced compared to HFA-GlcCer levels in *Cgt*^-/- ^mice (Figure 2A). Presence of NFA-GlcCer in *Fa2h*^-/-^/*Cgt*^-/- ^mice was better visible when the amount of lipids from these mice loaded was increased 4-fold compared to controls (Figure 2B). A similar reduction in GlcCer levels was seen when sphingolipids of purified CNS myelin were examined (Figure 3A). Densitometry revealed a reduction of NFA-GlcCer by more than 80% compared to HFA-GlcCer levels in *Cgt*^-/-^mice (Figure 3C). Analysis of peripheral nerves (sciatic nerve) revealed a reduction of GlcCer in *Fa2h*^-/-^/*Cgt*^-/- ^mice by about 60% compared to *Cgt*^-/-^mice (Figure 3B, D). MALDI-TOF mass spectrometry of hexosylceramides from *Cgt*^-/- ^and *Fa2h*^-/-^/*Cgt*^-/- ^mice (CNS myelin) demonstrated that cerebrosides in both genotypes mainly contained very long chain fatty acyl (VLCFA; C22:0, C24:0, and C24:1) residues (data not shown). The TLC analysis also revealed a strong increase in the very-long chain fatty acid (VLCFA)-containing sphingomyelin (SM; upper band) in *Fa2h*^-/-^/*Cgt*^-/-^, but also in *Fa2h*^-/-^/*Cgt*^+/- ^mice, when compared to *Cgt*^-/- ^and wild-type mice (note that *Fa2h*^-/- ^mice heterozygous for Cgt were used as a control in these experiments, because the number of *Fa2h*^-/-^/*Cgt*^+/+ ^mice were limited). Densitometry confirmed a 2-fold increase of sphingomyelin in CNS myelin of *Fa2h*^-/-^/*Cgt*^-/- ^mice compared to wild-type controls (and 60% increase in *Fa2h*^-/-^/*Cgt*^+/- ^mice; *Fa2h*^-/- ^were not analyzed) (Figure 3E). The increase of sphingomyelin in the PNS was about 50% in *Fa2h*^-/-^/*Cgt*^-/- ^mice (Figure 3F).

**Figure 4:**
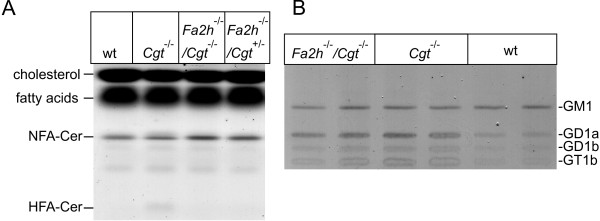
**TLC analysis of ceramides and gangliosides**. (A) TLC analysis of free ceramides in myelin confirmed the presence of HFA-ceramide in *Cgt*^-/- ^(lane 2) and its absence in *Fa2h*^-/-^/*Cgt*^-/- ^myelin (lane 3). NFA-ceramide levels were slightly increased in *Fa2h*^-/-^/*Cgt*^-/- ^mice (lane 3). Cer, ceramide. (B) The ganglioside pattern of purified myelin was not altered in *Fa2h*^-/-^/*Cgt*^-/- ^(lanes 1 and 2) compared to *Cgt*^-/- ^mice (lanes 3 and 4). The same amount of the major myelin ganglioside GM1 was found in both mouse lines, as well as in wild-type controls (lanes 5 and 6). The larger amount of the gangliosides GD1a, GD1b, and GT1b, which are major gangliosides of neuronal membranes, in the two mouse lines with less compact myelin are most likely caused by a higher proportion of axonal membranes in the myelin preparation.

In line with data published by Bosio et al. [18], HFA-ceramide was detectable in *Cgt*^-/- ^mice but not in mice of other genotypes (Figure 4A). However, in contrast to the 18-day-old *Cgt*^-/- ^mice analyzed by Saadat et al. [19], we did not observe a strong increase of ceramide in 4-week-old *Cgt*^-/- ^mice compared to wild-type controls. This might be due to the different ages examined.

To examine the possibility that GlcCer was replaced by gangliosides in myelin of *Fa2h*^-/-^/*Cgt*^-/- ^mice, gangliosides were isolated and analyzed by TLC. Although the total amount of gangliosides in the myelin fraction of *Fa2h*^-/-^/*Cgt*^-/- ^and *Cgt*^-/- ^mice was increased compared to wild-type, the major myelin ganglioside GM1 was unchanged and only gangliosides normally found in neuronal membranes (GD1a, GD1b, GT1b) were increased (Figure 4B). Most likely these gangliosides are derived from neuronal membranes and possibly reflect a higher proportion of neuronal membrane contaminations in the *Cgt*^-/- ^and *Fa2h*^-/-^/*Cgt*^-/- ^mice, with their significant reduced amounts of compact myelin. In line with this, Saadat et al. [19] showed a similar relative composition of major gangliosides in myelin preparation from mice deficient in *Ugcg *in myelinating cells. We therefore believe that myelin ganglioside levels in *Fa2h*^-/-^/*Cgt*^-/- ^mice were not increased compared to *Cgt*^-/- ^mice or wild-type controls. In summary, we conclude that HFA-GlcCer present in *Cgt*^-/- ^CNS myelin was replaced by only low amounts of NFA-GlcCer but larger amounts of NFA-sphingomyelin in *Fa2h*^-/-^/*Cgt*^-/- ^mice. Taken together, these results indicate that myelin hexosylceramides were mainly replaced by sphingomyelin in the absence of *Fa2h *expression. It should be noted that the increase of sphingomyelin and the decrease of hexosylceramides were more pronounced in CNS myelin than in the PNS. This might indicate differences between oligodendrocytes and Schwann cells in the trafficking or metabolism of sphingolipids.

### Myelination in *Fa2h*^-/-^/*Cgt*^-/- ^mice as compared to *Cgt*^-/- ^mice

**Figure 5:**
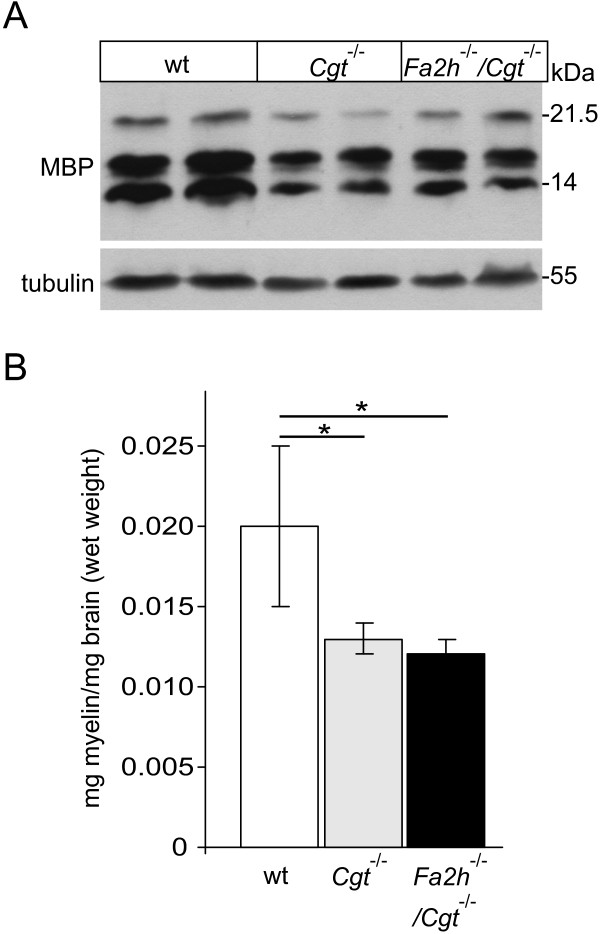
**Reduced levels of MBP and compact myelin in *Fa2h*^-/-^/*Cgt*^-/- ^and *Cgt*^-/- ^mice**. (A) Western blot analysis of 4-week-old wild-type (lanes 1 and 2), *Cgt*^-/- ^(lanes 3 and 4), and *Fa2h*^-/-^/*Cgt*^-/- ^mice (lanes 5 and 6) brain homogenates showed reduced MBP levels in *Cgt*^-/- ^and *Fa2h*^-/-^/*Cgt*^-/- ^mice. Shown is one representative out of three independent experiments, all of which gave comparable results. (B) The dry weight of purified myelin per mg brain wet weight isolated from *Cgt*^-/- ^and *Fa2h*^-/-^/*Cgt*^-/- ^mice (n = 3 per genotype) was significantly reduced compared to wild-type controls (*p < 0.05, t-test).

Myelin content in the brain of *Cgt*^-/-^, *Fa2h*^-/-^/*Cgt*^-/-^, and wild-type mice was examined by Western blot analysis of myelin basic protein (MBP) and by gravimetry of purified compact myelin. At the examined time point (4 weeks of age), MBP levels were reduced in *Cgt*^-/- ^mice compared to wild-type controls (Figure 5A), in agreement with our previous observations [20]. A comparable reduction of MBP was found in *Fa2h*^-/-^/*Cgt*^-/- ^mice. Accordingly, the amount of compact myelin isolated by sucrose gradient centrifugation was significant reduced in both, *Fa2h*^-/-^/*Cgt*^-/- ^and *Cgt*^-/- ^mice, compared to wild-type controls (Figure 5B), but there was no significant difference between the first two genotypes.

### Absence of GalCer and HFA-sphingolipids does not affect stability of CHAPS-insoluble membrane fractions

**Figure 6:**
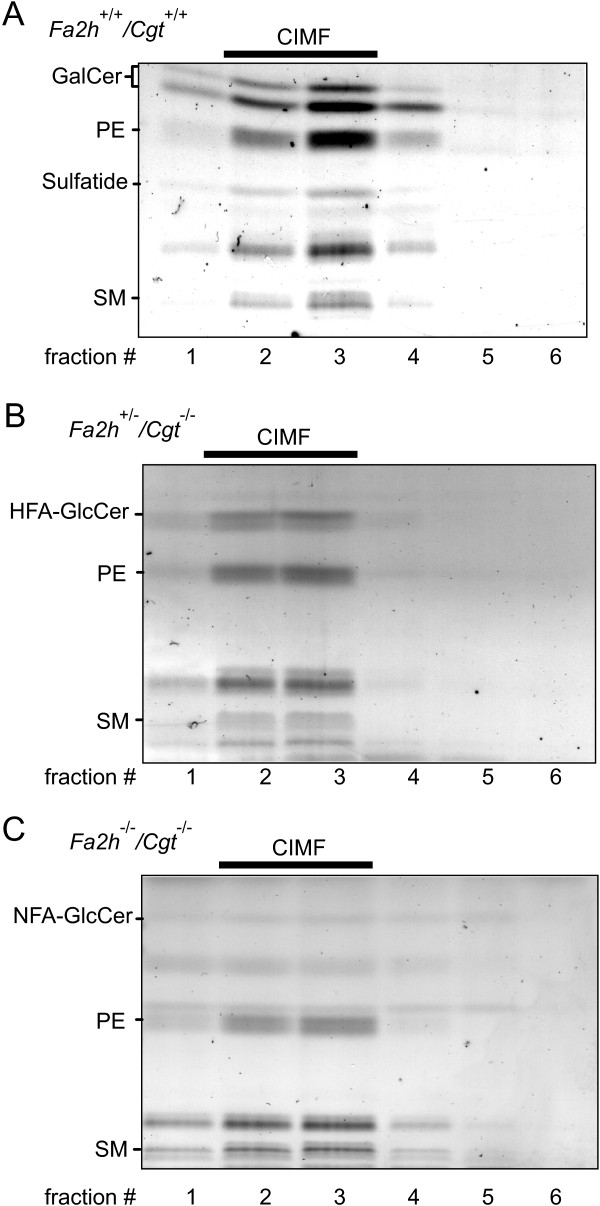
**CHAPS-insoluble membrane fractions in *Fa2h*^-/-^/*Cgt*^-/- ^myelin**. Myelin from wild-type (*Fa2h*^+/+^/*Cgt*^+/+^) (A), *Fa2h*^+/-^/*Cgt*^-/- ^(B), and *Fa2h*^-/-^/*Cgt*^-/- ^(C) mice isolated by sucrose density gradient centrifugation was treated with 20 mM CHAPS at 37°C for 30 min, followed by Optiprep density gradient centrifugation. Gradient fractions were collected from the top, and total lipids were extracted from all gradient fractions and separated by TLC. Most membrane lipids were present in the CHAPS-insoluble membrane fractions (CIMF), independent of the mouse genotype. Positions of lipid standards are indicated. These experiments have been done two times using two independent myelin preparations with similar results. PE, phosphatidylethanolamine; SM, sphingomyelin.

Compact myelin of wild-type mice is stable against extraction with the detergent CHAPS at low [21] and high temperature [15]. Data by Simons et al. [21] suggest that loss of HFA-GalCer in *Cgt*^-/- ^mice affects the association of the myelin proteolipid protein (PLP) with CHAPS-insoluble membrane fractions (CIMF), suggesting a role for (HFA-)GalCer in the formation or stabilization of CIMF. We have recently shown that the absence of HFA-sphingolipids does not affect stability of myelin CIMF [15]. However, in order to examine a possible synergistic effect of *Fa2h *and *Cgt *deficiency, purified myelin of *Fa2h*^-/-^/*Cgt*^-/- ^mice was subjected to CHAPS extraction at 37°C. Optiprep gradient centrifugations were performed and the fractions were examined for sphingolipid content, as described [15]. These experiments showed that myelin was resistant to CHAPS extraction irrespective of the genotype (Figure 6). Therefore, we conclude that neither GalCer nor HFA-sphingolipids are essential for stabilization of CIMF.

### Comparable myelination in *Fa2h*^-/-^/*Cgt*^-/- ^and *Cgt*^-/- ^mice

**Figure 7:**
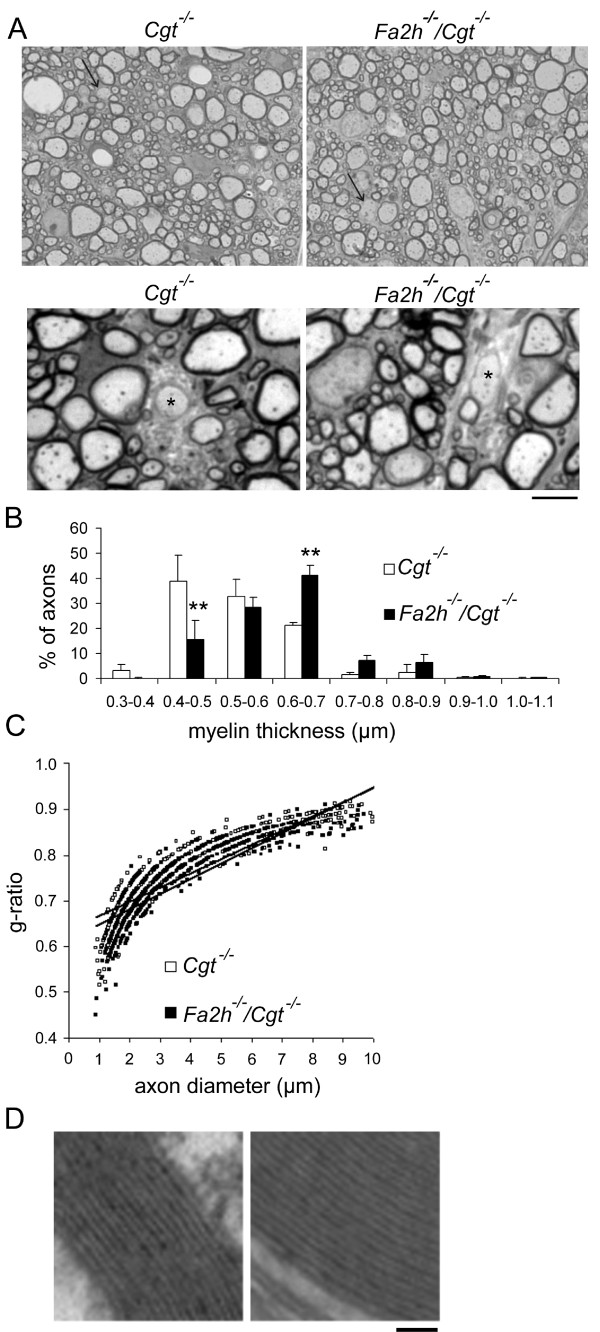
**Normal lamellar spacing in *Fa2h*^-/-^/*Cgt*^-/- ^myelin**. (A) Typical cross sections of spinal cords from *Cgt*^-/- ^and *Fa2h*^-/-^/*Cgt*^-/-^mice (two different magnifications are shown). Arrows indicate axons with thin or without myelin. Asterisks indicate demyelinated axons. Scale bar, 40 μm (upper panel) 10 μm (lower panel). Myelin thickness (B) and g-ratios (C) were determined in the spinal cord of three mice per genotype. Though there was a shift towards increased myelin thickness in *Fa2h*^-/-^/*Cgt*^-/- ^compared to *Cgt*^-/- ^mice (Data shown are the mean SD [n = 3 animals per genotype]; **p < 0.01, Chi-Square test), the g-ratios of myelinated axons were similar in *Cgt*^-/- ^and *Fa2h*^-/-^/*Cgt*^-/- ^mice. (D) Electron micrographs showing normal compact myelin in *Fa2h*^-/-^/*Cgt*^-/- ^mice (Magnification: 16,000×). Scale bar, 50 nm.

To evaluate the extent of myelin sheaths thickness, cross sections were obtained from the cervical spinal cord of 4-week-old mice. On each side of the midline, starting at the deep medial boundary of the ventral funiculus and extending ventrally and laterally, fibers of all calibers were included. In this unbiased sampling approach, the number of myelinated axons was unchanged in *Cgt*^-/- ^and *Fa2h*^-/-^/*Cgt*^-/- ^mice (179 ± 19 versus 194 ± 30). Only few axons in both mouse mutants displayed no myelin at this postnatal stage (Figure 7A). The mean axon diameter did not differ significantly between *Cgt*^-/- ^and *Fa2h*^-/-^/*Cgt*^-/- ^mice (3.88 μm versus 3.77 μm). The axons of *Fa2h*^-/-^/*Cgt*^-/- ^mice recruit inappropriately thick myelin relative to their absolute calibers resulting in an average myelin thickness of 0.60 ± 0.02 μm versus 0.53 ± 0.01 μm in *Cgt*^-/- ^(p < 0.01, Chi-Square test, n = 3) (Figure 7B). However, due to the relative small difference in myelin thickness, the g-ratios did not differ significantly between the two genotypes (Figure 7C). Furthermore, electron microscopy of myelinated axons revealed normal compact myelin in *Fa2h*^-/-^/*Cgt*^-/- ^mice (Figure 7D). These results demonstrate that a compact myelin sheath can be generated in the absence of GalCer and any 2-hydroxylated sphingolipids.

No obvious structural differences between oligodendrocytes of *Fa2h*^-/-^/*Cgt*^-/- ^and *Cgt*^-/- ^were observed by the histological or electron microscopic analyses, however, a detailed structural analysis was not performed, and therefore we cannot exclude subtle structural changes in *Fa2h*^-/-^/*Cgt*^-/- ^when compared to *Cgt*^-/- ^mice (e.g. in the paranodal region).

## Discussion

Although HFA-sphingolipids appear to be dispensable for the formation of compact myelin [15], they are essential for long-term myelin maintenance and may also play a role in glia-dependent axonal support. In the absence of GalCer in *Cgt*^-/- ^mice, compact myelin can be formed [3,4], which is, however, unstable, also suggesting a role of GalCer in myelin maintenance. We hypothesized that HFA-sphingolipids may also play a more subtle role in early postnatal development, which could be detectable on a *Cgt*^-/- ^background, where compact but less stable myelin is formed. However, we did not observe signs of a more severe phenotype in *Fa2h*^-/-^/*Cgt*^-/- ^mice compared to *Cgt*^-/- ^mice. At the behavioral level, *Cgt*^-/- ^and *Fa2h*^-/-^/*Cgt*^-/- ^mice were indistinguishable. Though there was a shift towards thicker myelin in *Fa2h*^-/-^/*Cgt*^-/- ^compared to *Cgt*^-/- ^mice, the g-ratios did not differ significantly between *Cgt*^-/- ^and *Fa2h*^-/-^/*Cgt*^-/- ^mice. Furthermore, myelin from *Fa2h*^-/-^/*Cgt*^-/- ^and *Cgt*^-/- ^mice was resistant towards extraction with CHAPS, as shown before for *Fa2h*^-/- ^and wild-type mice [15].

Unexpectedly, HFA-GlcCer present in *Cgt*^-/- ^mice was replaced by only low amounts of NFA-GlcCer in *Fa2h*^-/-^/*Cgt*^-/- ^mice but higher levels of NFA-sphingomyelin. This suggests that the GlcCer concentration in myelin is not critical and furthermore that elevated GlcCer levels in *Cgt*^-/- ^mice do not functionally compensate loss of GalCer. Accordingly, Saadat et al. [19] showed that deleting oligodendroglial glucosylceramide synthase (*Ugcg*) in *Cgt*^-/- ^mice did not reinforce the myelin phenotype. This demonstrates that upregulation of (HFA)-GlcCer in *Cgt*^-/- ^mice does not functionally compensate loss of GalCer in these mice. In *Ugcg*-deficient *Cgt*^-/- ^mice, HFA-GlcCer was partially replaced by HFA-sphingomyelin [19]. Our results demonstrate that HFA-sphingomyelin can be replaced by NFA-sphingomyelin without any obvious effect on the phenotype of *Cgt*^-/- ^mice. Thus, the significant up-regulation of HFA-sphingomyelin in *Cgt*^-/- ^mice is not a functional compensation.

The reason for the much lower NFA-GlcCer level in CNS myelin of *Fa2h*^-/-^/*Cgt*^-/- ^mice compared to the HFA-GlcCer level in *Cgt*^-/- ^mice is currently unknown. One possible explanation for the high HFA-GlcCer level and absence of HFA-gangliosides [9] in *Cgt*^-/- ^mice could be the inability of the responsible glycosyltransferases to use HFA-GlcCer as a substrate. However, presence of a high amount of HFA-gangliosides in other tissues [22] and in tumor cells [23] argues against this. An alternative explanation could be differential sorting of HFA- and NFA-GlcCer, as shown for polarized epithelial cell lines [24], or reduced half-life of HFA-sphingolipids.

Although structural changes at the paranodes of *Cgt*^-/- ^mice are clearly caused by sulfatide rather than GalCer deficiency [7,25], myelin appear to be much more stable in young adult *gal3st1*-deficient mice, whereas myelin maintenance is already affected in young *Cgt*^-/- ^mice [8], indicating additional function roles of HFA- and/or NFA-GalCer. Taken together, our results and those of Saadat et al. [19] strongly suggest that the upregulation of HFA-GlcCer and HFA-sphingomyelin in *Cgt*^-/- ^mice does not functionally compensate the loss of HFA-GalCer in these mice. This, however, does not exclude the possibility that GalCer could be functionally replaced by GlcCer, if the latter would be present at higher concentrations than in *Cgt*^-/- ^mice. The HFA-GlcCer concentration in *Cgt*^-/- ^mice is clearly below the concentration of HFA-GalCer in wild-type but also in heterozygous *Cgt*^+/- ^mice (which show a wild-type phenotype). There might be a relatively high threshold of hexosylceramide concentration in myelin to efficiently fulfill its role in myelin maintenance. This possibility could be tested using transgenic mice overexpressing *Ugcg *under control of an oligodendrocyte specific promoter, which to our knowledge are not available yet. Alternatively, the functional role of HFA- and/or NFA-GalCer may not be taken over by GlcCer or other glycolipids.

## Conclusions

Our data indicate that compact myelin can be formed with non-hydroxylated sphingomyelin as the predominant sphingolipid, though myelin maintenance is impaired. While the specific role of GalCer in myelin maintenance remains mysterious, our results suggest that the presence of HFA-GlcCer and HFA-sphingomyelin in *Cgt*^-/- ^mice does not functionally compensate the loss of HFA-GalCer.

## Methods

### Mice

*Fa2h*^-/- ^mice have been described previously [15]. Heterozygous *Cgt*^+/- ^mice (kindly provided by Dr. Brian Popko, University of Chicago) were crossed with *Fa2h*^-/- ^mice and double heterozygous *Fa2h*^+/-^/*Cgt*^+/- ^mice were interbred or bred with *Fa2h*^-/-^/*Cgt*^+/- ^mice to obtain mice of all possible nine genotypes. Genotyping was done using tail genomic DNA and the following oligonucleotides. *Fa2h *genotyping: 5'-GCTCTTCTTCAAGAGCCATCC-3', 5'-GTGCTGTACCTCAGCTGGTC-3'. 5'-ATTCGCAGCGCATCGCCTTCTATC-3', PCR products: 1045 bp for wild-type and 685 bp for the targeted allele; *Cgt *genotyping: 5'-TTACCAAGGAGTTCAGCAAACC-3', 5'-CCTCTCAGAAGGCAGAGACATTG-3', 5'-TCTGCACGAGACTAGTGAGACG-3', PCR products: 684 bp for wild-type and 820 bp for the targeted allele. All animal experiments followed internationally recognized guidelines and were approved by the Landesamt für Natur, Umwelt und Verbraucherschutz, Nordrhein-Westfalen, Germany.

### Lipid extraction and thin layer chromatography

Total lipid extracts were prepared from brains, sciatic nerves, or purified myelin (from 4-week-old animals) according to Bligh and Dyer [26]. In some experiments, phosphoglycerolipids were removed by mild alkaline hydrolysis as described [27]. In order to isolate gangliosides from purified myelin, lipids were extracted as described by Folch et al [28] and the ganglioside containing upper phase was desalted by reversed-phase chromatography using RP-18 columns (Merck, Darmstadt, Germany). Lipids were separated by thin layer chromatography (TLC) in one of the following solvent systems: (1) chloroform/methanol/water (65/25/4) for hexosylceramides and sphingomyelin, (2) chloroform/methanol/acetic acid (190/9/1) for ceramides, and (3) chloroform/methanol/0.22% CaCl_2 _(60/35/4) to separate gangliosides. HPTLC silica gel 60 plates (Merck) were used for all experiments. To visualize lipids, HPTLC plates were sprayed with cupric sulfate in aqueous phosphoric acid [27] followed by charring at 180°C for 5 min. Lipids were quantified by densitometry and the GalCer and GlcCer levels were normalized to cholesterol. Data are shown as the mean ± SD and were tested for statistically significant differences by ANOVA with post hoc Fisher's least significant difference (LSD) test using the program STATISTICA.

### Isolation of myelin

Compact myelin was isolated by sucrose gradient centrifugation as described by Norton and Poduslo [29], with minor modifications. Brains were homogenized in water using an Ultra-Turrax tissue homogenizer (IKA-Werke, Staufen, Germany). Aliquots of the homogenates were used for lipid extraction. The residual homogenates were adjusted to 10.5% (w/v) sucrose in 5 mM Tris-HCl (pH 7.4) and overlaid onto 10 ml of 30% (w/v) sucrose in 5 mM Tris-HCl (pH 7.4). After centrifugation (68,000×g, 50 min, 4°C) the enriched myelin fraction was recovered from the 10.5%/30% interphase, resuspended in 5 mM Tris-HCl (pH 7.4) and centrifuged at 68,000×g for 10 min. The resulting pellet was resuspended in 5 mM Tris-HCl (pH 7.4), centrifuged again, and the myelin was further purified by a second sucrose gradient centrifugation, followed by two washing steps in 5 mM Tris-HCl (pH 7.4). The final myelin pellet was resuspended in a small volume of water, lyophilized and stored at -80°C.

### Analysis of CHAPS insoluble membrane fractions

CHAPS insoluble membrane fractions (CIMF) of purified myelin were prepared by optiprep density gradient centrifugation of myelin samples treated with 20 mM CHAPS at 37°C, as described previously [15,21]. Six fractions of 350 μl each were removed from the top of the gradient and lipids were isolated from each fraction according to Bligh and Dyer [26], and analyzed by TLC as described above.

### MALDI-TOF mass spectrometry

MALDI-TOF MS of hexosylceramides was done as described [13].

### Western blotting

Brain samples were homogenized in 20 mM Tris-HCl (pH 8.0), 150 mM NaCl (TBS), containing 5 mM EDTA and 1 mM PMSF, using an Ultra-Turrax tissue homogenizer (IKA-Werke, Staufen, Germany). Homogenates were centrifuged at 1,000×g for 5 min and the supernatant was mixed with SDS-PAGE sample buffer containing 2-mercaptoethanol. Proteins were separated in 12.5%-polyacrylamide gels and transferred to nitrocellulose membranes by semi-dry blotting. Membranes were stained with rabbit anti-myelin basic protein (MBP; dilution 1:10,000; Millipore, Schwalbach, Germany) and mouse anti-alpha-tubulin (Developmental Studies Hybridoma Bank, University of Iowa). Bound secondary antibodies were detected by enhanced chemiluminescence as described [30]. Protein concentrations were determined with the Bio-Rad DC protein assay (Bio-Rad Laboratories, München, Germany) using bovine serum albumin as standard.

### Morphometrical analysis

Axon caliber and myelin thickness were measured on toluidine-stained semithin sections of the cervical spinal cord of 4-week-old mice. Axonal caliber was determined by the diameter of a circle of area equivalent to each axon. The g-ratio was determined by dividing the diameter of the axon by the diameter of the fiber (axon with myelin). Quantification of myelinated axons was performed with a semi-automatic program on the basis of AnalySIS using a light microscope (100× objective, BX60, Olympus). All morphometric measurements were conducted in a blinded manner using coded sections. Ultrathin sections (50 nm) were photomicrographed with an EM10 electron microscope (Zeiss, Germany) at a magnification of 16.000×.

## Authors' contributions

MM carried out the animal work, performed lipid and Western blot analyses and CIMF experiments. JJ carried out the morphometrical analyses and participated in writing the draft manuscript. CG participated in the design and coordination of the study. VG participated in the design and coordination of the study. ME conceived and designed the study, wrote the draft manuscript, and participated in the lipid and Western blot analyses. All authors read and approved the final manuscript.
